# Weight Regain After Discontinuation of GLP-1 and GIP/GLP-1 Receptor Agonists: A Systematic Review

**DOI:** 10.3390/metabo16100757

**Published:** 2026-10-09

**Authors:** Marco Marchetti, Giulia Campisano, Rosa Maria Paragliola, Davide Francomano, Valerio Cipolloni, Alessandro Conforti

**Affiliations:** 1 Departmental Faculty of Medicine, Unicamillus—Saint Camillus International University of Health Sciences, 00131 Rome, Italy; marco.marchetti@unicamillus.org (M.M.);; 2 Marchetti Professional Nutritionist Practice, 00131 Rome, Italy; giulia.campisano@gmail.com; 3 Department of Experimental and Clinical Medicine, Magna Graecia University of Catanzaro, 88100 Catanzaro, Italy; 4 Department of Orthopedics, University of Campania “Luigi Vanvitelli”, 80138 Naples, Italy; 5 Rheumatology Health Unit, ASL Roma 4, 00053 Civitavecchia, Italy

**Keywords:** GLP-1 receptor agonist, tirzepatide, semaglutide, liraglutide, discontinuation, weight regain, exercise, obesity, systematic review

## Abstract

Background: Weight regain and loss of cardiometabolic benefit commonly follow the withdrawal of glucagon-like peptide-1 receptor agonists (GLP-1RAs) and dual glucose-dependent insulinotropic polypeptide/GLP-1 receptor agonists, yet maintenance strategies are incompletely characterized. Methods: We conducted a systematic review reported according to PRISMA 2020 of primary studies in adults with overweight or obesity, with or without type 2 diabetes. PubMed/MEDLINE, Europe PMC and ClinicalTrials.gov were searched from inception to September 2026, supplemented by backward/forward citation searching and cross-checking of recent systematic reviews. Eligible reports quantified weight or cardiometabolic outcomes at least 12 weeks after planned or unplanned treatment cessation, or tested a post-cessation maintenance strategy. Randomized reports were assessed with RoB 2 and non-randomized reports with ROBINS-I. Owing to incompatible estimands, follow-up schedules, cointerventions and overlapping trial families, results were synthesized narratively without a new pooled estimate. Results: Eighteen reports representing 16 study families were included. Randomized withdrawal trials showed renewed weight gain after treatment cessation: +6.9% over 48 weeks after switching from semaglutide to placebo in STEP 4, and +14.0% over 52 weeks after switching from tirzepatide to placebo in SURMOUNT-4. In the STEP 1 extension, participants regained 11.6 percentage points over one year, approximately two-thirds of their prior loss, while several cardiometabolic measures moved toward baseline. Real-world estimates were smaller and more variable because discontinuation was often inferred from prescriptions, follow-up weights were missing, and many patients restarted or switched therapy. Cardiometabolic rebound was associated with the degree of weight regain, but clinical cardiovascular events were not evaluated. Evidence for maintenance strategies was sparse: one randomized post-treatment study supported supervised exercise, with the exercise-plus-liraglutide group retaining an additional 5.1 kg of weight loss one year after both interventions ended compared with liraglutide alone; a small pilot supported sitagliptin plus metformin after liraglutide in polycystic ovary syndrome. No eligible controlled trial isolated a specific diet after cessation. Conclusions: Weight regain is frequent after stopping GLP-1-based therapy, although magnitude varies by drug, design, follow-up and subsequent treatment. Structured exercise may help reduce regain and preserve lean mass, but evidence for dietary, behavioral, tapering or pharmacologic transition strategies remains limited. Discontinuation should therefore be planned as part of chronic obesity care, with explicit monitoring and shared decision-making, rather than presented as a predictable short course.

## 1. Introduction

Obesity is a chronic, relapsing disease in which biological, behavioral, social and environmental drivers persist after weight loss. Contemporary anti-obesity medications can produce clinically important reductions in weight and cardiometabolic risk factors, but benefits generally depend on ongoing treatment and access [1,2,3].

GLP-1RAs reduce energy intake through central satiety and appetite pathways and, variably, delayed gastric emptying; tirzepatide combines GIP and GLP-1 receptor agonism [4]. Their effects extend beyond the scale. Recent work has reviewed therapeutic and safety signals in ocular disease [5], and a six-month real-world cohort of people with knee osteoarthritis described improvements in pain, WOMAC physical function and low-grade inflammation during semaglutide treatment [6]. These observations reinforce that discontinuation should be evaluated across weight, glycemia and other health domains, while avoiding the inference that benefits demonstrated during treatment necessarily persist after withdrawal.

Randomized withdrawal trials and post-trial extensions consistently report renewed weight gain after GLP-1-based therapy is stopped [7,8,9,10,11,12]. Recent evidence syntheses have quantified average rebound [13,14], but combining reports without distinguishing trial family, drug, planned withdrawal, real-world treatment gaps and subsequent restarting risks double-counting and misleading precision. Moreover, the practical question—what can reduce regain after treatment ends—has received less direct study.

This systematic review therefore had three aligned objectives: (1) to quantify study-specific weight regain after the discontinuation of liraglutide, semaglutide, tirzepatide or another eligible GLP-1RA; (2) to describe cardiometabolic rebound, operationalized as deterioration from end-of-treatment values in HbA1c or fasting glucose, blood pressure, waist circumference, lipids or insulin (not incident cardiovascular events); and (3) to evaluate planned nutritional, behavioral, exercise or pharmacologic maintenance strategies initiated before, during or after cessation. We also distinguish primary outcome evidence from contextual reviews and expert clinical advice.

## 2. Materials and Methods

### 2.1. Review Design and Reporting

This was a systematic review of primary studies and is reported in accordance with PRISMA 2020 [15]. It is not an umbrella review: systematic and narrative reviews were used only for citation searching, interpretation and comparison, and their participant data were not extracted as independent evidence. The review was not prospectively registered, and no public protocol was available; this is acknowledged as a limitation.

### 2.2. Eligibility Criteria and Operational Definitions

Weight regain was defined as an increase in kg or percentage of body weight from the end-of-treatment assessment to follow-up; where authors reported the fraction of prior weight loss regained, that definition was retained. Cardiometabolic rebound was defined as worsening from the end-of-treatment value in at least one prespecified marker: HbA1c or fasting glucose, systolic or diastolic blood pressure, waist circumference, lipid measures, or insulin. When an eligible report did not provide a post-withdrawal value for a prespecified marker, that outcome was classified as not reported; absence of data was not interpreted as absence of rebound. Because the included studies did not assess incident myocardial infarction, stroke or cardiovascular death after withdrawal, the review does not equate marker rebound with cardiovascular events. The eligibility criteria and operational definitions are summarized in Table 1.

### 2.3. Information Sources and Search Strategy

PubMed/MEDLINE, Europe PMC and ClinicalTrials.gov were searched from inception to September 2026. Search concepts covered GLP-1-based agents, discontinuation/withdrawal and post-treatment weight or metabolic outcomes. No language, free-full-text or review-only filter was used. References and citing articles of eligible reports and recent systematic reviews were searched. The search was cross-checked against evidence syntheses that had independently searched Embase, CENTRAL and Web of Science [13,14]. Subscription-only databases were not directly searched by the present review team; this is reported transparently rather than representing indirect coverage as a direct search. The information sources, complete search strategies, and record counts are presented in Table 2.

### 2.4. Study Selection and Data Extraction

Records were deduplicated programmatically using DOI, registry identifier, PMID and normalized title. Initial electronic screening was followed by a complete second-pass verification of candidate records and full-text exclusion reasons against the prespecified criteria. Independent duplicate screening by two named reviewers was not completed; this limitation and the absence of a prospective protocol are stated explicitly. For each included report, a structured form captured study family, setting, design, population, sample size, agent/dose, treatment and withdrawal duration, comparator, maintenance intervention, weight and cardiometabolic outcomes, reinitiation/switching and funding. Reports from the same trial family were linked to prevent double-counting.

### 2.5. Risk-of-Bias Assessment

Randomized trials and randomized-origin extensions were evaluated at the level of the reported withdrawal outcome using the Cochrane RoB 2 domains: randomization, deviations from intended interventions, missing outcome data, outcome measurement and selection of the reported result [16]. Non-randomized studies were assessed with ROBINS-I across confounding, participant selection, exposure classification, deviations, missing data, outcome measurement and selective reporting [17]. Overall judgments followed each tool’s algorithms. Post hoc grouping by achieved regain was treated as non-randomized because those groups were not assigned. Ratings informed interpretation but were not converted to a numeric score.

### 2.6. Synthesis

A new meta-analysis was not undertaken because the reports used incompatible estimands (change from randomization, end of treatment or original baseline), different agents and exposure durations, follow-up ranging from 12 weeks to two years, planned versus inferred cessation, and variable restarting/switching. Several reports arose from the same trial families. We therefore present structured tabulation and descriptive plots without pooling; published meta-analyses are discussed as contextual corroboration rather than reanalyzed [13,14].

## 3. Results

### 3.1. Study Selection

**Figure 1:**
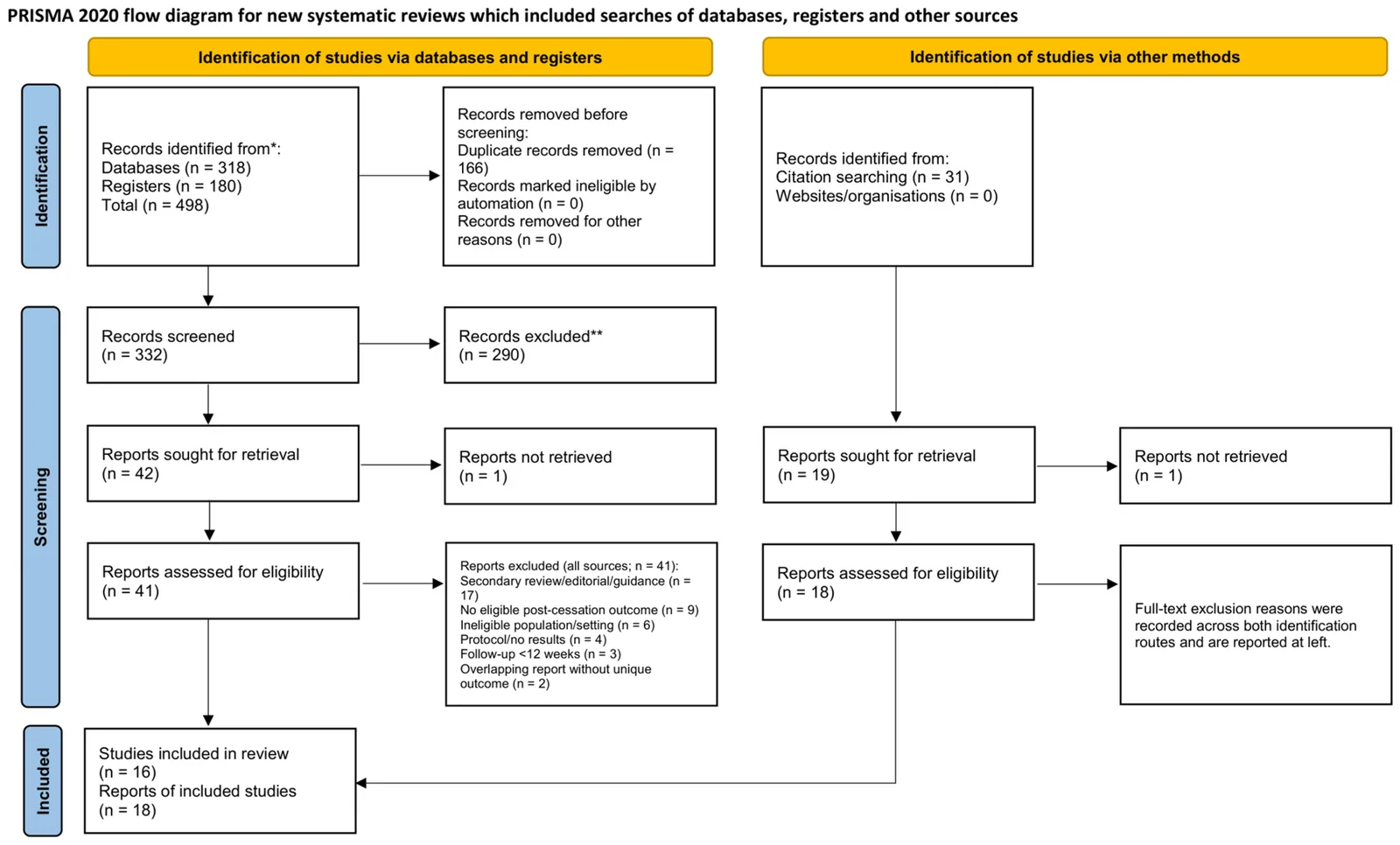
PRISMA 2020 flow diagram. Counts distinguish reports from study families; detailed exclusion categories sum to 41. * Records identified from databases and registers are detailed in Table 2. ** All records excluded at title/abstract screening were assessed manually; no automation tool was used.

The searches yielded 529 records (498 from databases/registers and 31 from citation searching). After the removal of 166 duplicates, 363 records were screened. Fifty-nine full-text reports were assessed; 41 were excluded, leaving 18 reports representing 16 study families (Figure 1). The most frequent full-text exclusions were secondary publications without unique primary data and the absence of an eligible post-cessation outcome.

### 3.2. Characteristics and Post-Discontinuation Outcomes

The evidence comprised randomized withdrawal trials, post-trial extensions, small prospective studies and large real-world cohorts. Populations included adults with obesity without diabetes, adults with type 2 diabetes, mixed cohorts, women with polycystic ovary syndrome and adults receiving antipsychotic medication. This breadth addresses the original mismatch between a non-diabetic PICOS statement and mixed populations in the results. Study characteristics and post-discontinuation outcomes are summarized in Table 3.

**Figure 2:**
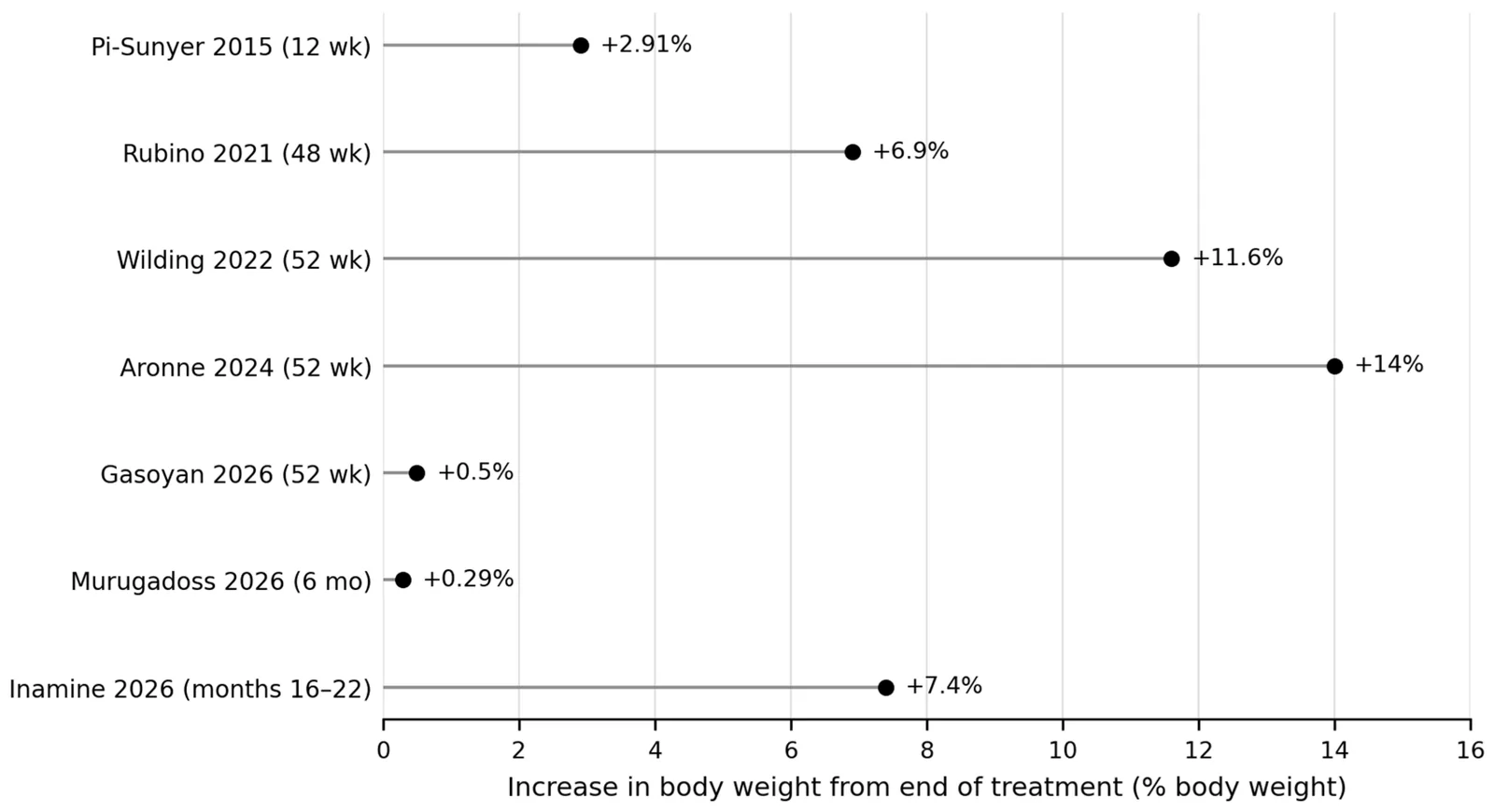
Study-reported increase in body weight after treatment cessation for reports using percentage-of-body-weight metrics. Values are descriptive and are not a meta-analysis; follow-up, design, estimand and exposure certainty differ.

Randomized withdrawal evidence produced the clearest causal contrast. In STEP 4, participants who switched from semaglutide to placebo gained 6.9% between weeks 20 and 68, whereas those continuing semaglutide lost an additional 7.9% [11]. In SURMOUNT-4, participants who switched from tirzepatide to placebo gained 14.0% between weeks 36 and 88, compared with a further 5.5% loss with continued tirzepatide; 16.6% versus 89.5%, respectively, maintained at least 80% of the initial weight loss [23]. In the STEP 1 extension, participants previously assigned semaglutide regained 11.6 percentage points during the off-treatment year—approximately two-thirds of their prior loss—but remained 5.6% below baseline [12]. These study-specific results support the direction of weight regain after withdrawal, while Figure 2 remains descriptive because the estimands and follow-up intervals are not directly poolable.

### 3.3. Cardiometabolic Rebound

STEP 1 participants experienced movement of blood pressure, glycemic and lipid measures toward baseline as weight was regained [12]. Across the included reports, waist circumference was reported less consistently than body weight and glycemia, and post-withdrawal insulin data were sparse. The SURMOUNT-4 post hoc analysis showed a dose–response pattern across achieved regain categories: systolic blood pressure rose by approximately 6.8, 7.3, 9.6 and 10.4 mmHg, and HbA1c by 0.14, 0.15, 0.27 and 0.35 percentage points across <25%, 25–<50%, 50–<75% and ≥75% regain groups, respectively; waist circumference and atherogenic lipid measures also worsened as the proportion of regained weight increased [26]. Because regain categories were not randomized, these associations cannot establish that regain caused each biomarker change. No included study was designed to assess post-withdrawal major adverse cardiovascular events. Thus, the available evidence demonstrates rebound in selected cardiometabolic markers, but not across the full prespecified panel, and not for clinical cardiovascular events.

### 3.4. Maintenance Strategies After Discontinuation

Table 4 shows that evidence for maintenance strategies is substantially less mature than evidence for weight regain. The strongest direct signal comes from S-LiTE, in which prior supervised exercise, particularly when combined with liraglutide, was associated with better one-year maintenance after both interventions ended, although attrition produced a high risk-of-bias judgment [20]. The sitagliptin-plus-metformin pilot suggested less short-term regain than metformin alone, but included only 24 women with polycystic ovary syndrome [9]. Findings for metformin plus lifestyle and exercise counseling were non-randomized and vulnerable to confounding [22,28], and no eligible controlled study isolated a post-cessation diet or behavioral program. Therefore, Table 4 identifies promising strategies and evidence gaps rather than an established standard of care.

### 3.5. Risk of Bias

**Figure 3:**
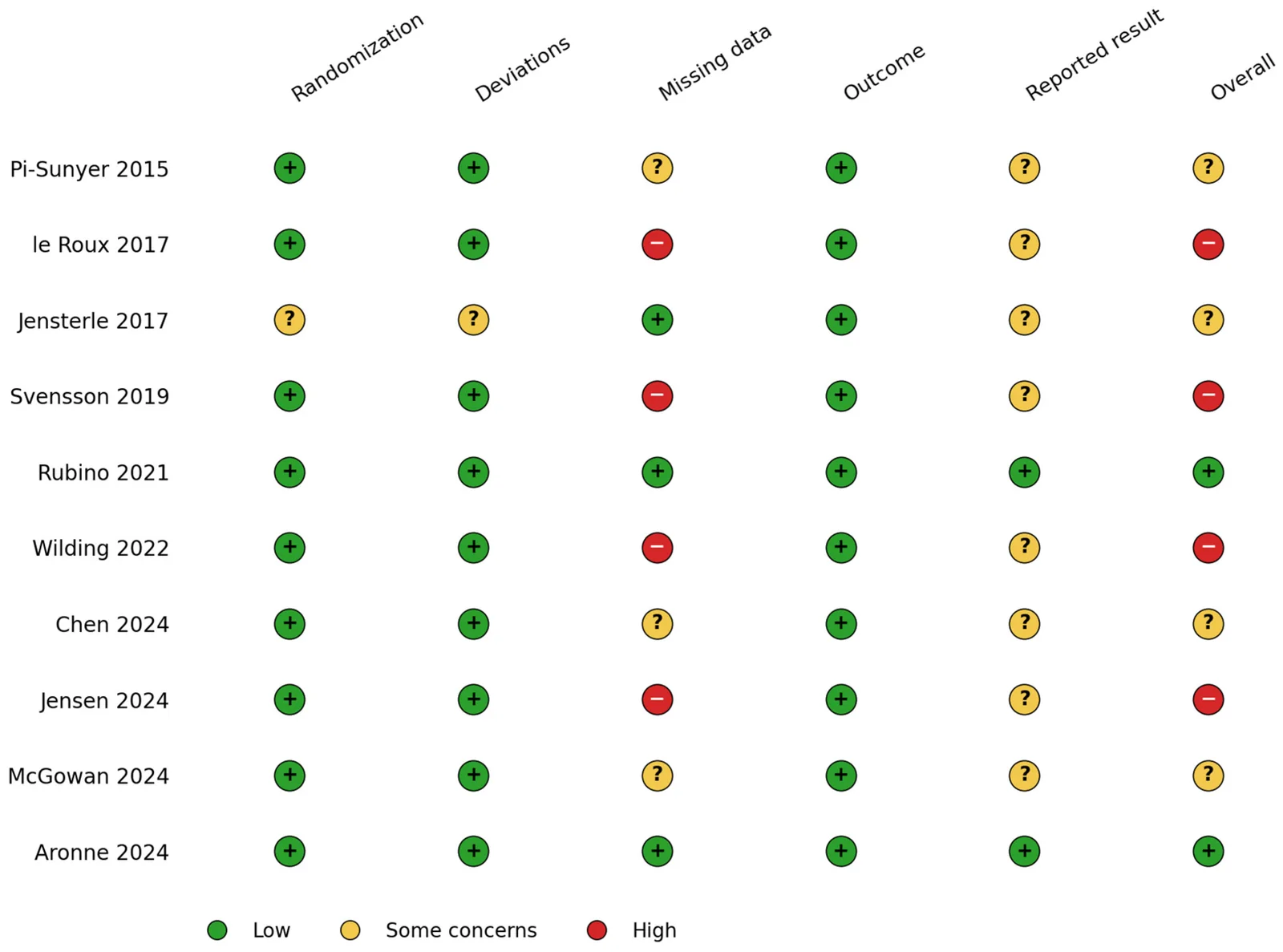
RoB 2 domain-level judgments for randomized and randomized-origin reports. ‘Selection’ denotes selection of the reported result. Sources: [7,8,9,10,11,12,19,20,21,23]. Symbols: green +, low risk; yellow ?, some concerns; red −, high risk.

**Figure 4:**
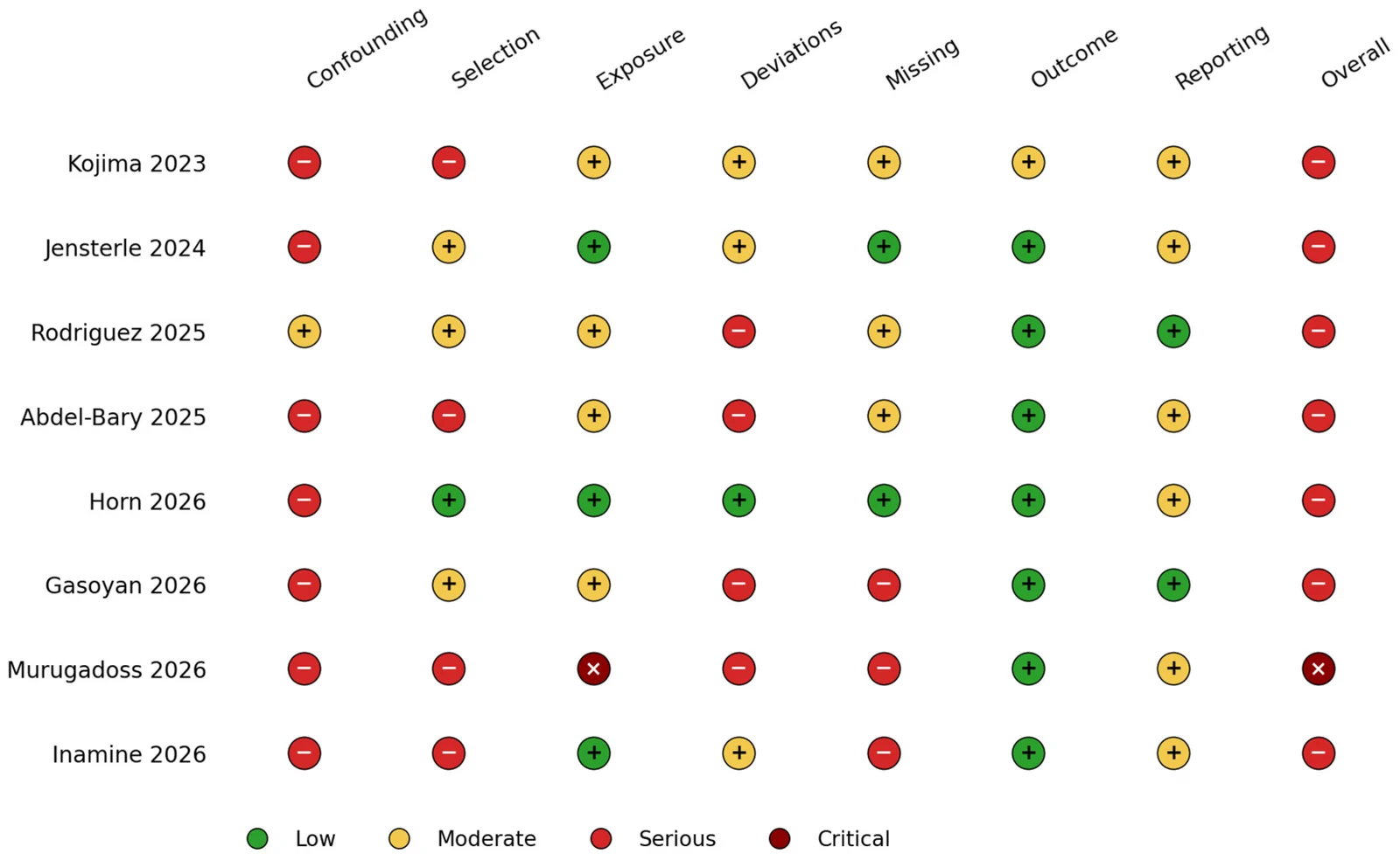
ROBINS-I domain-level judgments for non-randomized reports. Exposure denotes classification of discontinuation; outcome denotes outcome measurement. Sources: [18,22,24,25,26,27,28,29]. Symbols: green +, low risk; yellow +, moderate risk; red −, serious risk; dark red ×, critical risk.

Among the 10 randomized or randomized-origin reports in Figure 3, two—STEP 4 and SURMOUNT-4—were judged at low risk of bias for their randomized withdrawal contrasts [11,23], four raised some concerns, and four were judged at high risk. The low-risk contrasts provide the strongest basis for the causal conclusion that withdrawal leads to greater weight regain than continued treatment. Reports with some concerns or high risk contribute longer follow-up and additional outcomes, but attrition and selection into post-treatment follow-up reduce confidence in their numerical estimates. Figure 4 shows a still weaker evidence base among non-randomized reports: seven were judged at serious risk and one at critical risk. These studies provide useful information on routine-care discontinuation, restarting and cointerventions, but confounding, missing weights, participant selection and the uncertain classification of discontinuation prevent causal interpretation. The risk-of-bias assessments therefore affect the weight given to the findings: the low-risk randomized trials anchor the principal conclusion, whereas the non-randomized studies provide contextual rather than confirmatory evidence.

## 4. Discussion

### 4.1. Principal Findings

The most reliable evidence indicates that stopping semaglutide or tirzepatide causes clinically important weight regain relative to continued treatment [11,23]. Regain begins within months and can continue for at least one year, but it is neither uniform nor synonymous with immediate return to baseline. Trial extensions quantify biological and behavioral response under planned withdrawal; routine-care cohorts answer a different question because apparent discontinuers may restart, switch, undergo surgery or receive other weight-affecting treatments [24,27,28]. These designs should not be collapsed into a single unqualified rate.

The cardiometabolic objective also requires precision. Glycemia, blood pressure, waist circumference and lipid measures tended to worsen as weight was regained, and SURMOUNT-4 provides a graded association [12,26]. Post-withdrawal insulin evidence was insufficient to determine whether the same pattern occurred for that prespecified marker. However, the available outcomes are risk markers rather than observed cardiovascular events, and absence of a reported post-withdrawal value should not be interpreted as preserved benefit. The current evidence therefore supports the phrase ‘cardiometabolic marker rebound’, and not a demonstrated rebound in myocardial infarction, stroke or mortality.

If interpretation were restricted to the two low-risk randomized withdrawal trials, the conclusion would be narrower but clinically important: withdrawal of semaglutide or tirzepatide causes substantially more weight regain than continued treatment over 48–52 weeks [11,23]. These trials directly support the first review objective. They do not, by themselves, establish a universal regain percentage, effects beyond one year or comparative effectiveness across agents. They also provide no low-risk evidence for the third objective, because the evaluated maintenance strategies came from smaller or higher-risk reports. For the second objective, randomized trial data support deterioration in selected secondary cardiometabolic markers, but the detailed graded association between the amount of regain and cardiometabolic worsening derives from a post hoc, non-randomized analysis [26] and should be interpreted as associative.

Figure 4 further demonstrates that the newest real-world evidence is not necessarily the most reliable. These studies are valuable for describing discontinuation, reinitiation and routine-care cointerventions, but serious confounding, selection, missing weights and uncertain exposure classification preclude causal attribution. The critical judgment for Murugadoss et al. means that its numerical regain estimate should not be used to determine the magnitude of a withdrawal effect [28]. The observational findings are therefore used to assess generalizability and identify care pathways, not to override the randomized evidence.

### 4.2. How This Review Relates to Prior Reviews

Tzang et al. pooled 18 randomized trials (3771 participants) and reported a mean regain of 5.63 kg in obesity and 2.03 kg in type 2 diabetes, with HbA1c increases of 0.25 and 0.65 percentage points, respectively [13]. West et al. synthesized 37 medication-withdrawal studies (9341 participants) and estimated an average regain rate of roughly 0.4 kg/month, faster after newer incretin-based drugs [14]. Shah et al. emphasized clinical planning for weight and cardiometabolic consequences [30], and Quarenghi et al. reviewed randomized discontinuation evidence [31]. These reviews corroborate the direction of effect [32]. We did not count their study populations again; instead, we linked overlapping trial families and focused the new synthesis on study-specific estimands and intervention gaps.

### 4.3. Mechanisms: Plausible, Not Proven Mediators

Stopping a GLP-1-based agent removes its pharmacologic effects on appetite and energy intake. After weight loss from any cause, lower leptin, higher ghrelin and adaptive reductions in energy expenditure can favor regain [33,34]. Nevertheless, the included cessation studies generally did not measure these pathways as causal mediators. They should therefore be described as plausible general weight-loss physiology rather than proven drug-specific explanations for the observed rebound.

### 4.4. Exercise, Lean Mass and Sarcopenia

The S-LiTE follow-up provides the strongest direct signal that a structured exercise program begun during treatment may improve maintenance after liraglutide cessation [20,35]. Accordingly, resistance exercise and adequate aerobic activity may help reduce weight regain and preserve lean mass, but ‘essential’ overstates the present evidence. Loss of lean mass does not by itself establish sarcopenia. Current consensus defines sarcopenia primarily by low muscle strength, confirmed by low muscle quantity or quality, with physical performance indicating severity [36]. Future discontinuation studies should measure strength and function, not infer sarcopenia from body composition alone.

### 4.5. Nutrition and Behavioral Care: Evidence Versus Advice

Professional statements recommend screening for malnutrition risk, adequate protein and micronutrient intake, resistance exercise, hydration, smaller meals when gastrointestinal symptoms occur, and long-term behavioral support [37]. These are reasonable clinical-care recommendations during GLP-1 therapy, but no eligible controlled trial isolated a specific diet after cessation. This review therefore separates two claims: nutrition care is clinically prudent, whereas a particular post-cessation dietary prescription has not yet been shown to prevent regain. Patients should receive individualized advice from qualified clinicians, especially when diabetes therapy or gastrointestinal symptoms change.

### 4.6. Clinical and Research Implications

Because obesity is a chronic, relapsing disease, the possibility of weight regain and the potential need for long-term treatment should be discussed before treatment begins and revisited before any planned interruption [1,30,33]. When cessation is necessary, clinicians should document the reason, agree on an individualized follow-up plan and monitor weight and glycemia early; blood pressure, waist circumference and lipids should be reassessed according to baseline cardiometabolic risk [30]. These recommendations reflect chronic-disease management and the observed withdrawal evidence, but the review does not define a single monitoring schedule.

Structured, progressive physical activity—including resistance training when safe—may be offered to support weight maintenance and the preservation of lean mass [20,35,37]. Nutrition counseling should prioritize adequate protein and micronutrient intake, hydration, treatment tolerability and sustainable eating patterns [37]. However, these recommendations should remain clearly separated from claims of proven post-cessation efficacy, because no controlled trial identified a specific diet that prevents rebound after GLP-1-based therapy.

Research priorities follow directly from the evidence hierarchy and unresolved gaps. The low-risk randomized evidence establishes that withdrawal increases weight regain relative to continuation [11,23], but it does not determine whether tapering, lower-dose maintenance, switching to another agent or a structured lifestyle program can attenuate that regain [30]. Adequately powered randomized trials should compare these strategies, use harmonized definitions and assessment time points, and follow participants for at least 12–24 months. They should prespecify a core cardiometabolic panel—including glycemia, blood pressure, waist circumference, lipids and insulin—together with body composition, muscle strength, physical function and clinical cardiovascular outcomes. Real-world studies should model treatment restarting, switching, bariatric surgery and other weight-affecting medications as time-varying cointerventions, because failure to do so was a major source of bias in the current evidence.

### 4.7. Strengths and Limitations

This review has several strengths. It used explicit PICOS eligibility criteria, a multi-source search supplemented by citation searching, and trial-family linkage to reduce double-counting. Primary studies were separated from contextual reviews, risk of bias was assessed with design-appropriate tools, and heterogeneous estimands were not forced into an inappropriate pooled estimate. The synthesis also distinguishes cardiometabolic markers from clinical cardiovascular events and separates intervention evidence from clinical advice.

This review has several limitations. First, the updated search expanded substantially beyond the original single PubMed phrase, but direct searching was limited to PubMed/MEDLINE, Europe PMC and ClinicalTrials.gov; Embase, Scopus/Web of Science and CENTRAL were covered only indirectly through citation mapping and comparison with recent comprehensive reviews. Residual database and language-indexing bias is possible. Second, screening and extraction underwent a full second-pass verification but not independent duplicate review by two named investigators. Third, the review was not prospectively registered and had no public protocol. Fourth, clinical and methodological heterogeneity was substantial: agents, doses, treatment durations, definitions of cessation, estimands and follow-up differed, and several reports came from the same trial families. Fifth, extension cohorts had attrition, whereas real-world cohorts had missing weights, confounding, uncertain exposure classification and frequent treatment restarting or switching. Sixth, no new meta-analysis was appropriate, so between-study precision is descriptive. Finally, clinical cardiovascular events, muscle strength and rigorously tested post-cessation diet or behavior programs were largely absent. Potential publication bias and selective non-reporting of post-withdrawal outcomes cannot be excluded. Formal funnel-plot or small-study-effect assessments were not appropriate because no sufficiently homogeneous meta-analysis was conducted and too few studies contributed to any one comparable contrast.

## 5. Conclusions

Weight regain is common after GLP-1RA and GIP/GLP-1RA discontinuation, and the clearest randomized evidence shows substantially worse maintenance after withdrawal than after continued therapy. Glycemic, blood-pressure, waist and lipid benefits may also recede, but post-withdrawal cardiovascular events remain unstudied. Supervised exercise may help reduce regain and preserve lean mass; evidence for specific dietary, behavioral, tapering or transition regimens is insufficient. Discontinuation should be treated as a planned phase of chronic obesity care with monitoring, shared decision-making and explicit contingency plans for recurrence.

## Figures and Tables

**Table 1:** PICOS eligibility criteria and operational definitions.

PICOS Element	Operational Criterion
Population	Adults (≥18 years) with overweight or obesity, with or without type 2 diabetes or polycystic ovary syndrome. Mixed populations were eligible when an overweight/obesity subgroup was reported.
Intervention/exposure	Discontinuation of an eligible GLP-1RA or dual GIP/GLP-1RA after ≥12 weeks of treatment, whether withdrawal was randomized, planned at trial end or observed in routine care.
Comparator	Continued active therapy, placebo/withdrawal, an alternative maintenance intervention, or within-person end-of-treatment value. A comparator was not mandatory for descriptive cohorts.
Outcomes	Primary: change in body weight (kg, % or proportion of prior loss regained) ≥ 12 weeks after cessation. Secondary: HbA1c/glucose, blood pressure, waist, lipids, insulin, treatment restart/switching, lean mass or function.
Study design	Randomized withdrawal trials, post-treatment extensions, prospective/retrospective cohorts and case series with ≥5 participants. Reviews, editorials, guidelines, protocols without results and isolated case reports were excluded from the primary synthesis.
Maintenance strategy	A planned nutritional, behavioral, exercise or pharmacologic strategy temporally linked to discontinuation, with post-cessation follow-up.

**Table 2:** Information sources, search strategies and records identified. The asterisk (*) denotes the truncation wildcard used in the database search strings.

Source	Search as Run/Approach	Records
PubMed/MEDLINE	((GLP-1 receptor agonist OR glucagon-like peptide-1 OR semaglutide OR liraglutide OR tirzepatide OR exenatide OR dulaglutide) AND (discontinue * OR withdraw * OR cessat * OR stop * OR interruption) AND (weight regain OR rebound OR weight maintenance OR post-treatment OR cardiometabolic))	154
Europe PMC	TITLE_ABS:(GLP-1 OR semaglutide OR liraglutide OR tirzepatide) AND TITLE_ABS:(discontinue * OR withdraw * OR cessation OR stopped) AND TITLE_ABS:(weight OR regain OR rebound OR cardiometabolic)	164
ClinicalTrials.gov	Condition: obesity OR overweight; Other terms: GLP-1 OR semaglutide OR liraglutide OR tirzepatide; outcome/status fields screened for discontinuation, withdrawal, follow-up, regain or maintenance	180
Citation searching	Backward and forward citation searching of included reports and the recent systematic reviews by Tzang et al. and West et al. [13,14]	31

**Table 3:** Characteristics and results of included primary reports. *N* values refer to the reported analytic population where available. Reports sharing a trial family are identified to avoid double-counting. RoB 2 terms are used for randomized reports; ROBINS-I terms are for non-randomized reports.

Study/Family	Design	Population/*N*	What Was Done/ Duration	Post-Discontinuation Findings	Bias
Pi-Sunyer et al., 2015 [7] SCALE Obesity	RCT; randomized withdrawal component	Adults with overweight/obesity without diabetes; trial *n* = 3731; off-treatment subset ≈ 350	Liraglutide 3.0 mg for 56 wk; 12 wk off-treatment follow-up	After treatment stopped, mean regain was 2.91% (SD 3.01) of body weight over 12 wk.	Some concerns
le Roux et al., 2017 [8] SCALE 3-year	RCT extension; same trial family as [7]	Prediabetes and overweight/obesity; *n* = 2254 randomized	Liraglutide/placebo to 160 wk; 12 wk off-treatment follow-up	Weight increased during the off-treatment period in both groups; interpretation limited by attrition and selected long-term completers.	High
Jensterle Sever et al., 2017 [9]	Open-label pilot RCT	Women with PCOS after liraglutide; *n* = 24	Sitagliptin + metformin vs. metformin for 12 wk after liraglutide	Regain +0.9 ± 2.5 kg vs. +4.7 ± 2.7 kg (*p* < 0.001), supporting a pharmacologic transition signal.	Some concerns
Svensson et al., 2019 [10]	1-y follow-up of randomized trial	Antipsychotic-treated adults; follow-up *n* ≈ 46	Liraglutide stopped after 16 wk; 1-y follow-up	Some weight benefit persisted, but treatment-associated metabolic advantages diminished; attrition limits certainty.	High
Rubino et al., 2021 [11] STEP 4	Double-blind randomized withdrawal RCT	Adults with overweight/obesity without diabetes; 803 randomized after run-in; withdrawal *n* = 268	Semaglutide 2.4 mg for 20 wk then placebo vs. continued semaglutide to wk 68	Switching to placebo produced +6.9% weight change from wk 20–68 versus −7.9% with continuation.	Low
Wilding et al., 2022 [12] STEP 1 extension	Off-treatment extension of RCT	Extension cohort *n* = 327	Semaglutide/placebo for 68 wk; 52 wk off treatment	Semaglutide participants regained 11.6 percentage points—about two-thirds of prior loss—yet remained 5.6% below baseline. Several cardiometabolic markers moved toward baseline.	High
Kojima et al., 2023 [18]	Single-center case series	Adults with type 2 diabetes and obesity; *n* = 9	Tirzepatide for 52 wk; follow-up at 2, 4 and 6 mo after cessation	Early, dose-related weight regain and HbA1c re-elevation were observed; sample was very small.	Serious
Chen et al., 2024 [19]	Phase 3 RCT with post-treatment follow-up	Adults with overweight/obesity; randomized *n* = 427; follow-up analysis *n* = 221	Beinaglutide for 16 wk; 12 wk off-treatment follow-up	Authors reported a 0.78% weight-regain rate during follow-up; reporting metric was not directly comparable with STEP/SURMOUNT.	Some concerns
Jensen et al., 2024 [20] S-LiTE	Post-treatment follow-up of factorial RCT	Adults with obesity; *n* = 195 randomized; *n* = 109 at wk 104	1 y exercise, liraglutide, combination or placebo; then 1 y without study intervention	Combination retained 5.1 kg more loss than liraglutide alone at 1 y off treatment (95% CI 0.2–10.0 kg). Exercise reduced regain, but attrition was substantial.	High
McGowan et al., 2024 [21] STEP 10	Double-blind RCT with off-treatment follow-up	Obesity and prediabetes; *n* = 207	Semaglutide 2.4 mg for 52 wk; 28 wk off-treatment follow-up	Weight and glycemic benefits attenuated after cessation; the study was not powered for post-withdrawal clinical events.	Some concerns
Jensterle et al., 2024 [22]	Prospective observational cohort	Women with PCOS; *n* = 25	Semaglutide 16 wk, then metformin and lifestyle for 2 y	Mean weight 101→92 kg during semaglutide and 95 kg at 2 y: approximately one-third of loss regained; 84% remained below baseline.	Serious
Aronne et al., 2024 [23] SURMOUNT-4	Double-blind randomized withdrawal RCT	Adults with obesity without diabetes; 783 lead-in; 670 randomized; withdrawal *n* = 335	Tirzepatide for 36 wk then placebo vs. continued tirzepatide to wk 88	Withdrawal produced +14.0% weight change from wk 36–88 versus −5.5% with continuation; 16.6% vs. 89.5% maintained ≥ 80% of prior loss.	Low
Rodriguez et al., 2025 [24]	US electronic-health-record cohort	Adults with overweight/obesity with or without type 2 diabetes; *n* = 125,474	Routine-care semaglutide/tirzepatide; 1-y discontinuation and reinitiation	Discontinuation: 64.8% without vs. 46.5% with diabetes. Among discontinuers, reinitiation: 36.3% vs. 47.3%; greater regain predicted restart.	Serious
Abdel-Bary et al., 2025 [25]	Observational cohort	Adults after GLP-1RA discontinuation; *n* = 219	Routine-care discontinuation; 1-y follow-up	Substantial mean regain (approximately 7.3 kg) was reported, but confounding, selection and subsequent treatment were incompletely controlled.	Serious
Horn et al., 2026 [26] SURMOUNT-4 post hoc	Post hoc non-randomized analysis within withdrawal arm	Tirzepatide-withdrawal participants with data; *n* = 308	Participants grouped by proportion of prior loss regained	Greater regain was associated with larger increases in waist, SBP, HbA1c and atherogenic lipids; groups were achieved, not randomized.	Serious
Gasoyan et al., 2026 [27]	Multisite real-world cohort	Obesity *n* = 3321; type 2 diabetes *n* = 4617	Semaglutide/tirzepatide discontinuation; 1-y follow-up	19.6% restarted index therapy and 35.2% used another weight-affecting treatment. Among 3810 with weights, obesity mean +0.5% and diabetes −1.3%; selection and switching complicate interpretation.	Serious
Murugadoss et al., 2026 [28]	Real-world retrospective cohort	Adults with last semaglutide/tirzepatide prescription; *n* = 4182	6-mo follow-up after last recorded prescription	Mean +0.29%; 33.0% after semaglutide and 27.6% after tirzepatide had ≥2% regain. A confirmed-discontinuation subset was only *n* = 119; exposure misclassification was critical.	Critical
Inamine & Uesato, 2026 [29]	Single-center retrospective cohort	Obesity without diabetes; *n* = 104 (45 primary; 59 post-bariatric surgery)	Semaglutide cessation with late follow-up in a subset	95% of 22 regained by 2 mo and 83% of 12 by 6 mo; mean change shifted from −14.4% at month 16 to −7.0% at month 22. Late samples were very small.	Serious

**Table 4:** Direct evidence for maintenance strategies. Advice from professional statements is not presented as intervention efficacy evidence.

Strategy	Evidence Source	Design	Intervention	Finding	Interpretation
Supervised exercise	Jensen et al. [20]	Randomized intervention followed by 1-y off-treatment follow-up	Exercise initiated during weight loss, alone or with liraglutide	Prior exercise, particularly exercise + liraglutide, was associated with less regain and a more favorable body-composition trajectory than liraglutide alone.	Promising but limited by attrition and one study
Pharmacologic transition	Jensterle Sever et al. [9]	Small open-label pilot RCT	Sitagliptin + metformin vs. metformin after liraglutide	Less 12 wk regain with sitagliptin + metformin.	Very small, specific PCOS population
Metformin + lifestyle	Jensterle et al. [22]	Single-arm cohort	Metformin and lifestyle after short semaglutide course	About one-third of prior loss regained over 2 y; most remained below baseline.	No contemporaneous comparator
Exercise counseling	Murugadoss et al. [28]	Retrospective association	Counseling documented in routine care	More common among those without clinically relevant regain (26.2% vs. 14.7%; *p* = 0.04).	Confounding and critical exposure misclassification
Specific diet/behavior program	No eligible controlled study	—	No isolated post-cessation diet tested	Clinical nutrition advice exists for therapy tolerability and adequacy, but effectiveness for preventing post-cessation regain is unknown.	Evidence gap

## Data Availability

The search strings, screening counts, extraction fields, and risk-of-bias judgments supporting this review are available in the article and accompanying PRISMA documents.
